# Anti-allergic Activity of Stem Bark of *Myrica esculenta* Buch.-Ham. (Myricaceae)

**DOI:** 10.4103/0975-1483.62219

**Published:** 2010

**Authors:** KG Patel, NJ Rao, VG Gajera, PA Bhatt, KV Patel, TR Gandhi

**Affiliations:** *Department of Pharmaceutical Chemistry, Anand Pharmacy College, Shri Ram Krishna Seva Mandal Campus, Near Town hall, Anand - 388 001, Gujarat, India*

**Keywords:** Allergy, eosinophil, inflammation, *Myrica esculenta*, vascular permeability

## Abstract

Allergic diseases, such as allergic asthma, are hypersensitivity reactions initiated by immunological mechanisms. *Myrica esculenta* (*M. esculenta*) is known traditionally in Ayurveda to possess anti-asthmatic activity. The present investigation was undertaken to evaluate the effect of crude extract of stem bark of *M. esculenta* (Family Myricaceae, commonly known as Kaiphal) on experimental allergic reactions. Experimental models studied were allergic pleurisy and vascular permeability induced by acetic acid in mice. Pretreatment with *M. esculenta* (75 mg/kg and 150 mg/kg, p.o.) significantly inhibited the eosinophil accumulation (*P* < 0.01) respectively in the pleural cavity. *M. esculenta* (75 and 150 mg/kg, p.o.) significantly inhibited the rise in plasma exudation (57.12% and 59.77%, *P* < 0.01) induced by acetic acid in mice. These findings demonstrate that the crude extract from the stem bark of *M. esculenta* possesses antiallergic activity. This activity may be mediated by reducing the release of mediators such as histamine, inhibition of mast cell degranulation, and inhibition of eosinophil accumulation thereby preventing the release of cytokines and chemokines.

## INTRODUCTION

Allergic rhinitis and asthma, recognized as immediate-type hypersensitivity reactions, are characterized by increased responsiveness of the tracheobronchial tree to a variety of stimuli, which may be spontaneous, allergen-related, or drug-induced, and the primary pathophysiological abnormality being bronchial wall inflammation leading to airway narrowing.[1–3]

The prevalence and severity of allergic asthma has been steadily increasing over past 20 years together with the number of reported cases of fatal asthma, and it affects up to 10% of the population of most developed countries.[4,5] The disease statistics clearly necessitates the increasing need for drugs targeting the mechanisms involved in eosinophil and neutrophil activation and accumulation, anti-IgE therapy for the management of asthma. Glucocorticosteroids are the only drugs currently available that effectively reduce airway inflammation in asthma.[6] As a result there is high prevalence of usage of complementary and alternative medicines for treatment of this disease.[7]

*Myrica esculenta* Buch. Ham. (Syn. *Myrica sapida*, Family Myricaceae, commonly known as Kaiphal) is known traditionally in Ayurveda for the treatment of asthma and bronchitis.[8,9] Preliminary studies performed at our laboratory has established the efficacy of *M. esculenta* in decreasing bronchial hyper-responsiveness and bronchoconstriction through systematic studies on bronchoalveolar lavage, acetyl choline and histamine-induced bronchospasm, and lung cytology in guinea pigs.[10,11] The pathophysiology of asthma involves multiple factors leading to hyper-responsiveness and bronchoconstriction, such as mast cell degranulation,[12] neurogenic dysfunction,[13] involvement of T-lymphocytes and eosinophils,[14,15] altered immunosuppressive macrophages,[16,17] overproduction of proinflammatory cytokines,[18,19] immunoglobulins (IgE, IgG, and IgD)[20] and plasma protein exudation.[21–23] Therefore, the present work aims to prove the antiallergic activity based on studies focusing upon inflammation of the airway.

## MATERIALS AND METHODS

### Plant

Aerial parts of *M. esculenta* were obtained from commercial supplier of Ahmedabad. The plant was identified and authenticated by Dr. Minoo Parabia, Head and Professor, Department of Bioscience, Veer Narmad South Gujarat University, Surat, Gujarat. A voucher specimen of plant was deposited in the herbarium of the Department of Bioscience, Veer Narmad South Gujarat University, Surat, Gujarat.

### Preparation of the plant extract

The aerial parts reduced to coarse powder were macerated with ethanol for 48 h, filtered, and filtrate was evaporated under reduced pressure to obtain a dry extract. The ethanolic extract of *M. esculenta* (referred to as EtMe) was stored in cool and dry place and used for pharmacological evaluation.

### Reagents

Egg albumin and aluminum hydroxide hydrate gel (alum) were purchased from S. D. Fine Chem Limited, India. Freund’s complete adjuvant emulsion, and Evans blue were purchased from HiMedia, India. All other chemicals used were of analytical grade.

### Animal protocol

Swiss albino mice of either sex housed in standard conditions of temperature (22 ± 2°C), relative humidity (55 ± 5%), and light (12 hrs light/dark cycles) were used. Animal studies were approved by the Institutional Animal Ethics Committee (protocol no 8004), as per the guidelines of committee for the purpose of control and supervision of experiments on animals (CPCSEA).

### Vascular permeability induced by acetic acid[24,25]

Swiss albino mice were randomly allocated to four groups each containing six animals. Group I (model control), Group II (standard), Group III (EtMe), and Group IV (EtMe) received saline, indomethacin (20 mg/kg), EtMe (75 mg/kg), and EtMe (150 mg/kg) orally, respectively. Thirty minutes later, mice received an intravenous injection of 0.5% Evans blue saline solution (0.1 ml/10 g body weight) and an intraperitoneal injection of 0.6% acetic acid (10 ml/kg). After 20 min, the dye that leaked into the peritoneal cavity was collected by lavaging with 10 ml distilled water and was transferred to 10 ml volumetric flask through glass wool. To each flask, 0.1 ml of 0.1 N sodium hydroxide solution was added and volume made upto the mark with distilled water followed by measurement of absorbance at 610 nm (Shimadzu Spectrophotometer).

### Allergic pleurisy

Swiss albino mice were randomly allocated to five groups each containing six animals: Group I (normal control), Group II (model control), Group III (standard), Group IV (EtMe), and Group V (EtMe). Active sensitization of Swiss albino mice was achieved with a subcutaneous injection of Freund’s complete adjuvant emulsion (100 μL) containing egg albumin (100 μg). Fourteen days later, mice were challenged with an intrathoracic injection of egg albumin (50 μL, 12.5 μg/cavity).[26,27] Briefly, an adapted needle was inserted into the right side of the thoracic cavity of egg albumin sensitized animals to permit the intrapleural administration of egg albumin diluted in sterile pyrogen free saline (50 μL). At 24 h after the stimulus, mice were anesthetized and their thoracic cavities were rinsed with 1 mL phosphate buffer saline containing 10 mM EDTA, pH 7.4. Total leukocyte counts were made with an automated cell counter (Cell Dyn 3200SL). Differential cell counts were made by light microscopy stained with Leishman’s stain. Groups IV and V animals fasted overnight and received EtMe (75 mg/kg) and EtMe (150 mg/kg) orally 1 h before stimulation. Group II animals were similarly treated with vehicle alone. In Group III, dexamethasone was given intraperitoneally (2 mg/kg) 24 and 1 h before stimulation.

### Statistical analysis

The results of various studies were expressed as mean ± SEM and analyzed statistically using one-way ANOVA with Dunnett *post hoc* test to find out the level of significance. Data were considered statistically significant at P < 0.05.

## RESULTS

### Effect of EtMe on vascular permeability induced by acetic acid

Pretreatment with EtMe (75 mg/kg) and EtMe (150 mg/ kg) significantly inhibited the rise in plasma exudation (57.12% and 59.77% inhibition) induced by acetic acid in mice as compared to the control group. Indomethacin (20 mg/kg) also significantly inhibited the exudation (70.69%) [Table 1].

**Table 1:** Effect of EtMe on vascular permeability induced by acetic acid

Group	Evans blue concentration (mg/ml)	% Inhibition
Control	1.474 ± 0.121	-
Standard (Indomethacin, 20 mg/kg)	0.432 ± 0.14*	70.69
EtMe (75 mg/kg)	0.632 ± 0.151*	57.12
EtMe (150 mg/kg)	0.593 ± 0.222*	59.77

Statistical analysis by one way ANOVA with Dunnett *post hoc* test; values are mean ± SEM; n = 6 in each group; significantly different from control group

* *P* < 0.05

### Effect of EtMe on allergic pleurisy

Twenty-four hours after the intrathoracic injection of egg albumin, an intense accumulation of total and differential leukocytes was observed in sensitized as compared to the control group. Dexamethasone pre-treatment (2 mg/ kg, i.p.) significantly inhibited the influx of total and differential leukocytes as compared to the sensitized group. The oral pretreatment with EtMe (75 mg/kg and 150 mg/ kg, po) markedly inhibited the eosinophil accumulation as compared to the control group and this inhibition was selective for decrease in the number of eosinophils. Conversely, the number of total leukocytes, lymphocyte, monocytes, and neutrophils was not significantly affected on oral pre-treatment with EtMe (75 mg/kg and 150 mg/kg, po) [Table 2].

**Table 2:** Effect of EtMe on cell counts in allergic pleurisy

Group	Total leukocyte	Neutrophil	Lymphocyte	Eosinophil	Monocyte
Control	3575411	1857.75±291.39	1567.25±169.71	65.5±14.06	83.25±17.95
Sensitized	5225±383.78*	3072.75±264.34*	2002±108.44	145±27.54*	111.75±5.63*
Dexamethasone (2 mg/kg)	2550±413.32@	1273.5±202.37@	1182.25±187.97@	32.75±9.3@	61.5±20.36
EtMe (75 mg/kg)	4425±968.14	2630.5±761.26	1679.5±219.2	51.75±8.87	63.25±12.92
EtMe (150 mg/kg)	4423±259.41	2319.25±277.2@	1938±160.67	67±14.29@	100.75±15.96

Statistical analysis by one-way ANOVA with the Dunnett *post hoc* test; values are mean ± SEM, n = ± in each group; significantly different from control group;

* *P* < 0.05 (one-way ANOVA); significantly different from sensitized group;

@ *P* < 0.05 (one-way ANOVA with the Dunnett *post hoc* test)

## DISCUSSION

Allergic processes are complex disorders in which inflammatory and immunological mechanisms are involved. Bronchial asthma is one such allergic process characterized by inflammation of the airways usually accompanied by increased vascular permeability, resulting in plasma exudation. Plasma protein leakage has been implicated to play a role in the induction of a thickened, engorged, and edematous airway wall, resulting in the airway luminal narrowing correlating with bronchial hyper-reactivity and airway inflammation.[28,29] It is also possible that the plasma exudates may readily pass the inflamed mucosa and reach the airway lumen through leaky epithelium, thus compromising epithelial integrity and reducing ciliary function and mucus clearance.[30,31] It is also reported that increased vascular permeability is associated with bronchial inflammation and airway hyper-responsiveness in a murine model of asthma.[32,33] Consistent with these observations, we have found that amounts of plasma extravasation were greatly enhanced in a murine model of allergic airway disease. Interestingly, administration of EtMe inhibited vascular permeability increase thereby inhibiting vascular leakage resulting in decreased airway inflammation and airway hyper-responsiveness.

Additionally, an important causative factor of allergic disease is eosinophilic inflammation.[34,35] The triggering and regulation of eosinophil accumulation in allergic inflammation depend on the release of cytokines and chemokines such as interleukin-4 (IL-4), IL-5 and CCL11/eotaxin in response to an antigen challenge.[36,37] Among the C-C chemokines, CCL11/eotaxin, an eosinophil-specific chemoattractant, is one of the most important mediators of allergic inflammation, with a potent and selective effect in mobilizing eosinophils from bone marrow to the blood.[37–40] To participate in the allergic inflammatory response, eosinophil must migrate from the circulation to the airway.[41,42] Circulating eosinophils migrate to the airways by the phenomenon of cell rolling, through interaction with P-selectin, and eventually adhere to the endothelium through the binding of integrins to adhesion proteins, vascular cell adhesion molecule 1 (VCAM 1) and intercellular adhesion molecule 1 (ICAM 1). As eosinophils enter the matrix of the membrane, their survival is prolonged by IL-5. The cytoplasmic granules of eosinophils have a crystalloid core of major basic protein and a matrix of eosinophil cationic protein, eosinophil-derived neurotoxin, and eosinophil peroxidase that are released on activation. Major basic protein and eosinophil cationic protein have profound cytotoxic effects on the airway epithelium,[43] and for this reason, eosinophils are often regarded as the primary effector cells in asthma. The modulation of eosinophil accumulation is one of the main targets for the discovery of anti-allergic compounds because of its potential tissue damaging effects. Above facts were further substantiated by observing the potential effect of EtMe which produced a pronounced inhibition of eosinophil accumulation in allergic pleurisy, hence decreasing allergic inflammation.

## CONCLUSION

Overall data from our experimental studies suggest that ethanolic extract of *M. esculenta* possesses significant anti-allergic and anti-inflammatory activity and may be useful in the treatment of allergic disorders such as allergic asthma and allergic rhinitis by decreasing bronchial hyper-responsiveness.

## References

[1] (1987). Standards for the diagnosis and care of patients with chronic obstructive pulmonary disease (COPD) and asthma. This official statement of the American Thoracic Society was adopted by the ATS Board of Directors, Nov 1986. Am Rev Respir Dis.

[2] Pride NB (1992). Asthma. Definition and clinical spectrum. Br Med Bull.

[3] Jeffery PK (1992). Pathology of asthma. Br Med Bull.

[4] O’byrne PM, Postma DS (1999). The many faces of airway inflammation-Asthma and Chronic obstructive pulmonary disease. Am J Respir Crit Care Med.

[5] Giembycz MA (2000). Phosphodiesterase 4 inhibitors and the treatment of asthma-where are we now and where do we go from here. Drugs.

[6] Barnes PJ, Pedersen S (1993). Efficacy and safety of inhaled corticosteroids in asthma. Report of a workshop held in Eze, France, October 1992. Am Rev Respir Dis.

[7] Salib RJ, Drake-Lee A, Howarth PH (2003). Allergic rhinitis: Past, present and the future. Clin Otolaryngol Allied Sci.

[8] Kirtikar KR, Basu BD (1999). Indian Medicinal Plants.

[9] Nadkarni’s KM (2002). Indian Materia Medica.

[10] Patel KG, Patel KV, Shah JH, Monpara KB, Gandhi TR (2008). Evaluation of the effect of Myrica sapida on bronchoconstriction and bronchial hyperreactivity. Pharmazie.

[11] Patel KG, Bhalodia PN, Patel AD, Patel KV, Gandhi TR (2008). Evaluation of bronchodilator and antianphylactic activity of Myrica sapida. Iran Biomed J.

[12] Chapel H, Haeney M, Mishbah J, Snowden N (1999). Essentials of Clinical Immunology.

[13] Busse WW, Calhoun WF, Sedgwick JD (1993). Mechanism of airway inflammation in asthma. Am Rev Respir Dis.

[14] Corrigan CJ, Kay AB (1992). T-cell and eosinophils in the pathogenesis of asthma. Immunol Today.

[15] Kline JN, Hunninghake GW (1994). T-lymphocyte dysregulation in asthma. Proc Soc Exp Biol Med.

[16] Poulter LW, Janossy G, Power C, Sreenan S, Burke C (1994). Immunological/Physiological relationships in asthma: Potential regulation by lung macrophages. Immunol Today.

[17] Spiteri MA, Poulter LW (1991). Characterization of immune inducer and suppressor macrophage from normal human lung. Clin Exp Immunol.

[18] Barnes PJ, Liew FY (1991). Nitric oxide and asthmatic inflammation. Immunol Today.

[19] Najam FI, Giasuddin AS, Shembesh AH (2001). Tumour necrosis factors in childhood Asthma. Indian J Pediatr.

[20] Najam FI, Giasuddin AS, Shembesh AH (1999). Immunoglobulin isotypes in childhood asthma. Indian J Pediatr.

[21] Lee KS, Kim SR, Park HS, Jin GY, Lee YC (2004). Cysteinyl leukotriene receptor antagonist regulates vascular permeability by reducing vascular endothelial growth factor expression. J Allergy Clin Immunol.

[22] Lee YC, Kwak YG, Song CH (2002). Contribution of vascular endothelial growth factor to airway hyperresponsiveness and inflammation in a murine model of toluene diisocyanate induced asthma. J Immunol.

[23] Thurston G, Rudge JS, Ioffe E, Zhou H, Ross L, Croll SD (2000). Angiopoietin-1 protects the adult vasculature against plasma leakage. Nat Med.

[24] Whittle BA (1964). The use of changes in capillary permeability in mice to distinguish between narcotic and nonnarcotic analgesics. Br J Pharmacol Chemother.

[25] Yeşilada E, Küpeli E (2002). Berberis crataegina DC. root exhibits potent anti-inflammatory, analgesic and febrifuge effects in mice and rats. J Ethnopharmacol.

[26] Sampaio AL, Rae GA, Henriques MM (2000). Role of endothelins on lymphocyte accumulation in allergic pleurisy. J Leukoc Biol.

[27] Henriques MG, Weg VB, Martins MA, Silva PM, Fernandes PD, Cordeiro RS (1990). Differential inhibition by two hetrazepine PAF antagonists of acute inflammation in the mouse. Br J Pharmacol.

[28] Thurston G, Rudge JS, Ioffe E, Zhou H, Ross L, Croll SD (2000). Angiopoietin-1 protects the adult vasculature against plasma leakage. Nat Med.

[29] Van de Graaf EA, Out TA, Roos CM, Jansen HM (1991). Respiratory membrane permeability and bronchial hyperreactivity in patients with stable asthma. Effects of therapy with inhaled steroids. Am Rev Respir Dis.

[30] Persson CG (1996). Epithelial cells: Barrier functions and sheddingrestitution mechanisms. Am J Respir Crit Care Med.

[31] Foster WM, Langenback EG, Bergofsky EH (1982). Lung mucociliary function in man: Interdependence of bronchial and tracheal mucus transport velocities with lung clearance in bronchial asthma and healthy subjects. Ann Occup Hyg.

[32] Lee KS, Kim SR, Park HS, Jin GY, Lee YC (2004). Cysteinyl leukotriene receptor antagonist regulates vascular permeability by reducing vascular endothelial growth factor expression. J Allergy Clin Immunol.

[33] Lee YC, Kwak YG, Song CH (2002). Contribution of vascular endothelial growth factor to airway hyperresponsiveness and inflammation in a murine model of toluene diisocyanate induced asthma. J Immunol.

[34] Wark PA, Gibson PG (2003). Clinical usefulness of inflammatory markers in asthma. Am J Respir Med.

[35] Bochner BS, Busse WW (2004). Advances in mechanisms of allergy. J Allergy Clin Immunol.

[36] Robinson D, Hamid Q, Bentley A, Ying S, Kay AB, Durham SR (1993). Activation of CD4+T cells, increased TH2-type cytokine mRNA expression, and eosinophil recruitment in bronchoalveolar lavage after allergen inhalation challenge in patients with atopic asthma. J Allergy Clin Immunol.

[37] Palframan RT, Collins PD, Williams TJ, Rankin SM (1998). Eotaxin induces a rapid release of eosinophils and their progenitors from the bone marrow. Blood.

[38] Jose PJ, Griffiths-Johnson DA, Collins PD, Walsh DT, Moqbel R, Totty NF (1994). Eotaxin: A potent eosinophil chemoattractant cytokine detected in a guinea pig model of allergic airways inflammation. J Exp Med.

[39] Rothenberg ME, Luster AD, Leder P (1995). Murine eotaxin: An eosinophil chemoattractant inducible in endothelial cells and in interleukin 4-induced tumor suppression. Proc Natl Acad Sci U S A.

[40] Baggiolini M, Dewald B, Moser B (1997). Human chemokines: An update. Annu Rev Immunol.

[41] Bochner BS (1997). Cellular adhesion and its antagonism. J Allergy Clin Immunol.

[42] Wardlaw AJ (1999). Molecular basis for selective eosinophil trafficking in asthma: A multistep paradigm. J Allergy Clin Immunol.

[43] Corrigon CJ (1994). Immunological aspects of asthma: Implications for future treatment. Clin Immunol Ther.

